# Observations on the Illusions of the Insane, and on the Medico-Legal Question of Their Confinement

**Published:** 1833-10

**Authors:** 


*Observations on the Illusions of the Insane, and on the Medico-Legal Question of their Confinement.* Translated from the French of M. ESQUIROL, Mèdecin en Chef de la Maison Royale de Charenton, &c., by WILLIAM LIDDELL. London, 1833. 8vo. pp. 89.

WE confess that these two pamphlets have rather disappointed us. From a physician of Esquirol's great and deserved reputation, we certainly should have expected something more philosophical than this little essay on the Illusions of the Insane. We hoped to find some attempt at elucidating the manner in which these hallucinations gradually arise, at first but slightly clouding reason, but ultimately overwhelming it entirely; and we also looked for instructions as to the method of scaring away these visionary horrors, by educating the senses of the patient, and teaching him to correct the mistaken conclusions of one by the evidence of the others. But of this there is little or nothing. Still, cases selected by a great physician from his actual experience must always have some practical value; and we shall therefore quote a few from the twenty-seven with which M. Esquirol has favoured us. He divides them into two sections; the first consisting of those cases in which the illusions arose from internal, and the second of those in which the illusions depended on external, sensations. The following are in the first section.

"Internal sensations, produced by disturbance of the organic sensibility, often excite the illusions of the insane. The skin of some insane persons is dry, parched, rough, and burning, and badly performs its functions. These patients are indifferent to extremes of temperature. M. Pinel speaks of a maniac who filled his hands with snow, and rubbed his chest with it in ecstacy.

"1*st Observation.* The famous Térouane de Méricourt lived ten years at the Salpêtrière, in a state of madness. She used to throw two pails of water on her bed every morning and evening, and lie down immediately afterwards. I have seen her break up the ice to procure water from the fountains. Other insane persons feel such an irritation of the skin, that they fancy they are struck, and almost killed, by the slightest contact; and that poisons and burning substances are thrown upon them, &c. We have, at Charenton, a mad woman, who calls out, whenever she is touched, even with the point of the finger, 'You hurt me; do not strike me, do not strike me.'

"2*d Obs.* An artillery officer, twenty-seven years of age, of a sanguineous temperament, and of a strong and athletic form, was seized with an intermittent fever, during the Prussian campaign. They made him swallow a large glass of brandy, into which they had mixed the gunpowder of two cartridges. He became mad immediately, and tore up every thing that fell in his way, linen, wearing apparel, and bedclothes; they were obliged to let him sleep upon straw. Feeling himself pricked he placed the straw in a ring, leaving in the centre an empty space, which he occupied: he now moved his head in every direction, blowing incessantly upon the straw which surrounded him, and screaming from time to time, as if to drive away menacing objects. This symptom continued night and day for more than three weeks. It was then discovered that he mistook the straws for the beaks of birds of prey that had wounded him. He blew upon the straw, and screamed, in order to frighten away those annoying animals. Subsequently the same patient had new illusions. Soon after he retired to bed he tore it in pieces, and threw the straw of his paillasse, by handfuls, out of the chamber window, which was closed by blinds; and spoke from time to time as if he had been addressing horses. The noise of the persons whom he heard walking was taken by him for the footsteps of horses coming to the window as if to a rack. As the straw was stolen as fast as it was thrown out, it continued to keep up the illusion.

"The pains which insane persons feel in the different regions of the body are so many causes of illusion to them.

"3*d Obs.* Mlle. **** at eighteen years of age, enjoyed good health, although still irregular. Soon after the events of 1815, she experienced a fixed pain on the top of her head, and very soon persuaded herself that she had a worm in her cranium, which was devouring her brain. The sight of copper almost made her faint, and her friends were obliged to remove from her apartments every thing that was covered with gilding. She consented to walk with the greatest reluctance, because the dust raised by the pedestrians appeared to her filled with oxide of copper. Nothing could persuade her to touch a gilt candlestick, nor the cock of a fountain. After many months of unsuccessful treatment I was called to her. She was thin, her skin was discoloured, and she was very irritable. She sometimes refused to eat, slept badly, and had constipation of the bowels; she spoke at one time of her repugnances with liveliness, and at another time in tears. I endeavoured to gain her confidence by flattering her fancies, and by assuring her that I would destroy the worm which was the cause of all her complaints, if she had the courage to let me perform an operation, which would not be very painful to her. I so well succeeded in persuading her, that after one of my visits she made an incision herself with a penknife on her head. As soon as she saw the blood run she fainted. I was sent for immediately, and on my arrival I found the patient, who had recovered her recollection, very desirous that I should perform the operation of which I had so long spoken. The young lady's courage kept up that of her friends, who consented to the employment of the means which I had proposed. M. Bigot, the medical attendant of the family, made a crucial incision of more than two inches in length over the part affected, and allowed the blood to flow. We shewed the patient a small piece of fibrin, which we assured her was the insect that had been the cause of her suffering so long. An issue was made in the middle of the incision, and kept there for three months, when the fixed pain, illusions, and fears of verdigris, all disappeared together." (P. 5.)

We have not the original before us, but we would venture to conjecture that there is a mistranslation in the third case; for the words "enjoyed good health, though still irregular," have no very precise meaning. Esquirol probably says "quoiqu'elle n'était pas encore reglée;" *i. e.* although the catamenia had not yet appeared.

These fancied evils have been sometimes removed by humouring the patient, and giving a local habitation to the airy nothings of his imagination; as in the first of the following instances:

"*7th Obs.* Ambrose Paré relates that he cured an hypochondriacal patient, who fancied that he had frogs in his stomach, by giving him a purgative, and introducing by stealth little frogs into the pan which was to receive the alvine evacuations.

"I opened the body of an insane woman at the Salpêtrière, who said she had an animal in her stomach. She had a cancer of that viscus." (P. 10.)

The illusions depend on external sensations in those cases in which the lunatic takes pebbles for diamonds, or clouds for armies, &c. The following are some of our author's examples:

"*14th Obs.* A general officer, forty-six years old, of a nervous temperament, marrying, passed from a life of great activity to a state of uninterrupted tranquillity. About a year afterwards he became jealous: the jealousy increased, and the persons whom he received at his house, even his best friends, he accused of being the seducers of his wife. On several occasions he wished to fight with them, and followed them into his house sword in hand. After several months he was brought to Paris, where his uneasiness increased; for he mistook the cries in the streets for abuses addressed to him. He ran into some of the apartments of the hotel where he lived, to demand satisfaction of his imaginary rivals. At length, being unable to contain himself any longer, he wished to terminate his existence, and, asking one of his companions to give him some poison, he put his affairs in order: having made his will, he swallowed with transport a harmless draught which his friend presented to him. After some hours, not feeling the effects of the poison, he became enraged against his friend, who had deceived, betrayed, and ridiculed him. The general was confided to my care. A few days afterwards we walked together to St. Cloud. During our promenade he interrupted me several times in the midst of a very connected conversation, saying, 'Do you hear how they repeat the words *coward*, *jealous*, &c.' This illusion was produced by the noise of the leaves, and the whistling of the wind among the branches of the trees, which appeared to him well-articulated sounds; and although, I believed, I had each time combated it with success, the illusion returned whenever the wind agitated the trees anew.

"15*th Obs.* I had under my care a young lady, whom the most trifling noise terrified, especially during the night. The footsteps of a person walking, however lightly, made her shudder; the wind caused her to tremble; and the noise that even she herself made by moving in bed frightened her, and obliged her to get up, and utter cries of distress. I procured rest for this panophobist by keeping a light in her bedroom, and obliging a person to sit up with her all night.

"The sense of sight is more productive of illusions, in a state of health, than the other senses, because it is in more frequent communication with external objects. Illusions of sight are also very common amongst the insane, and give rise to resemblances which provoke raving, and almost always augment the disease. So that a relation or friend is mistaken for a stranger, or an enemy, of whom they have had reason to complain.

"16*th Obs.* A young married man was in a state of fury whenever he saw a woman leaning on a man's arm, being convinced that it was his own wife. I took him to the theatre, at the commencement of his convalescence; but, as soon as a lady entered the saloon, accompanied by a gentleman, he became agitated, and called out eagerly several times, 'That is she! that is she!' I could hardly help laughing, and we were obliged to retire.

"17*th Obs.* A lady, twenty-three years of age, afflicted with hysterical madness, used to remain constantly at the windows of her apartments during the summer. When she saw a beautiful cloud in the sky, she screamed out 'Garnerin, Garnerin, come and take me!' and repeated the same invitation until the cloud disappeared. She mistook the clouds for balloons sent up by Garnerin.

"A cavalry officer imagined the clouds which he saw to be an army, led by Bonaparte, to make a descent upon England.

"Insane persons often collect pebbles and fragments of glass, which they fancy precious stones, diamonds, or objects of natural history, and which they preserve with the greatest care." (P. 16.)

Panophobist *(panophobe)*, and Lypemania *(lypemanie)*, are words with which M. Esquirol has enriched the lingua medico-barbara: the former signifies one who is afraid of everything; the latter is synonymous with melancholia.

It is not uncommon to see patients who are afraid that their adviser is making them the subjects of some critical experiment; but, in the following case, a lady was afraid of actually becoming an experimental instrument.

"27*th Obs.* A lady, much reduced by a confinement, and by being bled to overcome an attack of madness, experienced obstinate constipation, for which I prescribed injections. Notwithstanding her illness, she wished to administer them herself; but she had scarcely taken the syringe in her hands when she threw it down with horror. The same circumstance occurred several times; and she has assured me since, that the syringe appeared so heavy that she thought it was filled with mercury, and that they wished to make a barometer of her body." (P. 26.)

M. Esquirol draws the following conclusions from the whole body of cases:

"1st. That illusions are caused by internal and external sensations.

"2d. That they are the result of the sentient extremities, and of the reaction of the nervous centre.

"3d. That they are as often caused by the excitement of the internal as by that of the external senses.

"4th. That they cannot be confounded with hallucinations (visions), since, in the latter cases, the brain only is excited.

"5th. That illusions lead the judgment astray respecting the nature and cause of the impressions actually received, and urge the insane to acts dangerous to themselves and to others.

"6th. That sex, education, profession, and habits, by modifying the reaction of the brain, modify also the character of the illusions.

"7th. That illusions assume the character of the passions, and of the ideas which govern the insane.

"8th. That reason dissipates the illusions of the man of sound mind, whilst it is not powerful enough to destroy those of the insane.

"If by observation I have been able to elucidate a psycological phenomenon but little appreciated, although common in delirium; if the facts which I have related throw some light upon the still obscure history of the aberrations of the understanding; or if they furnish therapeutic views applicable to the treatment of mental diseases, these observations will not be entirely without interest." (P. 26.)

In the first section of the remaining essay, our author enters at considerable length into the reasons which make it necessary to confine the insane; but we shall pass over these, as we do not think it worth while to give the proof of a proposition which nobody denies. Esquirol himself says, "All English, French, and German physicians, who have devoted themselves to the study of mental diseases, recommend the confinement of the insane, and are unanimous as to the utility of this means of cure." (P. 33.) The cases, however, brought forward to illustrate this self-evident proposition are well told and instructive in other points of view; we shall therefore quote a few of them.

"*1st Obs.* A merchant, fifty-five years of age, of a strong constitution, although of a lymphatic temperament, mild and gentle in his disposition, father of a numerous family, and who had acquired a considerable fortune in business, experienced some domestic troubles, not sufficiently serious, however, to affect any one of a resolute character.

"About a year ago he formed a large establishment for one of his sons, and shortly afterwards became very active, and expressed (contrary to his usual habits) the delight which he felt at his increasing prosperity. He was also more frequently absent from his warehouse on business than usual; but, notwithstanding these trifling changes, neither his family, nor any of his friends or neighbours, suspected any disorder in his reason. One day, whilst he was from home, a travelling merchant brought to his house two pictures, and asked fifty louis for them, which he said was the price agreed upon by a very respectable gentleman, who had given his name and address. His sons sent away both the pictures and the seller. On his return, the father did not mention his purchase; but the children began the conversation, alluding to the roguery of the merchant, and their refusal to pay him. The father became very angry, asserting that the pictures were very beautiful, that they were not dear, and that he was determined to purchase them. In the evening the dispute became warmer; the patient flew into a passion, uttered threats, and at last became delirious. On the next day he was confided to my care. His children, frightened at their father's illness, and alarmed at the purchase which he had made, looked through their accounts; and great was their astonishment at seeing the bad state of their books, the numerous blanks which they presented, and the immense deficiency of cash! This irregularity had existed more than six months. Had this discussion not taken place, one of the most honourable mercantile houses would have been compromised in a few days; for a bill of exchange, of a considerable amount, had now become due, and no means had been taken to provide for it." (P. 33.)

"*3d Obs.* M ****, twenty-seven years of age, of a sanguineous temperament, subject to headach, was attacked with a fit of madness, whilst riding on horseback when the weather was very hot. He was picked up on the road by some friends of his family, who confined him to his room until the arrival of his relations. He fancied he had fallen into the hands of thieves, because, when he entered his friend's house, they put his horse into the stable, and took charge of his portmanteau. After using all sorts of efforts and violence to recover his liberty, he set fire to the house, in order by that means to escape from those whom he mistook for thieves.

"I once had under my care a madman, who, to escape from an asylum in which he was confined, set fire to his bed, hoping to burn the house, and to effect his escape during the confusion occasioned by the fire." (P. 43.)

"*7th Obs.* A young man, twenty-one years of age, being melancholy for some days, was taken into the country by his companions, to divert his thoughts. During dinner an explosion of furious madness took place, without any apparent cause; he loaded his friends with abuse, called them scoundrels, and endeavoured to strike them. He was confined, and intrusted to my care. After three months' illness he recovered. On the decline of his disorder the sight of one of his friends sometimes produced agitation and even frenzy. When the cure was complete, he declared, that whilst at dinner with his companions the wine appeared to him of a horrible taste, and he fancied they had poisoned him.

"*8th Obs.* An emigrant, forty-six years old, of a sanguineous temperament, and of a peremptory character, after a long train of misfortunes, was arrested, but soon afterwards restored to his family. This circumstance threw him into despair, followed by an attack of madness, which continued for two months. During his delirium he saw and spoke of nothing but gendarmes, prisons, chains, &c. After this attack he remained melancholy and hypochondriacal. During the following year, without any fresh provocation, he became suddenly mad, and on the day afterwards was confided to my care. Although the delirium was general, with agitation, he spoke frequently, as during his first attack, of prisons, soldiers, &c. This delirium was evidently influenced by the remembrance of the arrest which had brought on the first attack. Whenever I went near the patient I addressed him in a friendly manner, familiarly offered him my hand, and reverted to the attentions I had paid him the year before. Dissipate your uneasiness, I often said to him, for you may depend upon my care; you are not obliged to remain, as there is nothing to prevent your going out whenever you please. On the fourth day I finished my usual exhortations with these words, hastily spoken, 'Let us take a walk!' The patient wished to follow me without his clothes; but I begged him to dress himself, and we went out. We had scarcely walked a dozen steps when he was able to exchange some coherent phrases with me; and before we returned to the house he had recovered the entire use of his faculties." (P. 53.)

"Having proved the necessity of confinement," says M. Esquirol, "it remains for me to shew its utility." It seems strange that this should remain, as we should have thought that whatever is necessary must be useful: it would appear however that, by "necessary," our author means necessary for the well-being of society; and, by "utility," the patient's own advantage. The following cases are intended to show the good effects produced by confinement.

"14*th Obs.* Mademoiselle de B***, twenty-seven years of age, tall, of a neuro-sanguineous temperament, and of a lively but sweet disposition, was tenderly attached to her mother, whom she had never left. After a somewhat violent disagreement she became sad; her menses became irregular, and at the expiration of two months she was seized with a fit of madness. During her delirium she conceived a great aversion towards her mother, and loaded her with reproaches and abuse. At times she tore her clothes, broke the furniture, uttered violent screams, and wished to leave her home. With this general delirium hysterical symptoms manifested themselves. A month afterwards she was confided to my care. Delirium was now general, with libidinous language; she became desirous of the company of men, and spoke against her mother. Many times in the course of the day her face became animated, her eyes sparkled, her speech was quick, and her loquacity continual. Convulsions came on, with constrictions about the throat; she screamed, flew into a passion, rolled upon the ground, &c. After seven months she experienced so violent an attack of hysteria, that her life appeared to me in danger for many hours. With the calm which followed this attack the delirium seemed to have diminished, and appeared only occasionally on the following days. After the expiration of a fortnight, mild small-pox made its appearance. As soon as the desquamation had ceased, the young lady returned to the bosom of her family, as reasonable and as amiable as before her illness. She continued quite well until the same period of the following year, when she experienced loss of sleep, reproached her mother, spoke much, became agitated, and left her bed in order to be better heard by her mother, who tried in vain to calm her, and to persuade her to lie down. Frightened at these fresh occurrences, her mother took post horses, four hours after the fresh attack, came to Paris, and confided her to my care. She spoke much, especially against her mother; ate little, and appeared tormented with thirst. In the evening she perceived the absence of her mother, and asked me where she was. 'Your mother has left,' I said to her, 'and you will remain with us until your health be re-established.' The countenance of the young lady immediately changed; instead of being animated she became sorrowful, her loquacity ceased, and she passed a tranquil night, although without sleep. On the following morning she appeared ashamed, shed tears, regretted the absence of her mother, and expressed a desire to return to her family. It is easy to perceive that the attack was cut short; for on the third day after her admission she received a visit from her mother, and obtained a promise that she should be allowed to return with her the next time she came. After the expiration of twelve days she returned to her family.

"*15th Obs.* M. N*****, at fifty-six years of age, of a very nervous temperament, and of a spare habit, having experienced great reverses in his political situation, gave himself up to study, and enervated his mind by too earnest application. At the beginning of winter he was attacked with monomania, and was intrusted to my care. His loquacity was interminable: he wrote incessantly, and was full of the desire to purchase into the funds, although he had great landed property, and had never entered into any such speculation before. After six months' attention at home, he was recommended to travel; which, in three months, confirmed the happy termination of this first attack. Four years afterwards, at the same time of the year, the beginning of winter, he came home, and told his wife, in a very satisfied manner, that he had just bought public stock to a very considerable amount. His wife, who had perceived for some days before that her husband was agitated, and had had less sleep than usual, persuaded him to travel. On the very next morning they set out on their journey, the purchase of stock was forgotten, and in a few days he recovered his health." (P. 56.)

Now it appears to us, that if Mademoiselle de B*** had been married, and that ill-used gentleman, M. N****. had been allowed to employ his own money as he liked, M. Esquirol would have lost two good patients, and this essay two brilliant examples. "From what precedes," says our author, after narrating a number of other cases, "I am led to the following conclusions:

"The insane ought to be confined:

"1. For their own security, for that of their families, and for the maintenance of public order.

"2. To remove them from the influence of the external causes which have produced their disorder, and may be likely to protract it.

"3. To overcome their resistance to curative means.

"4. To submit them to a regimen appropriate to their situation; and,

"5. To make them resume their moral and intellectual habits." (P. 71.)

But, after all this plainsailing, now comes the storm. We have to settle the knotty point of who shall be the judge of insanity: interested relations, or money-making keepers of madhouses? "Confinement having for its first object the privation of liberty, ought not the authorities to interfere in an act of so much importance? Yes, without doubt; but to conclude from this that all the insane should be subject to interdiction, would be erroneous." (P. 79.) That is to say, shall no man be confined in a lunatic asylum till a court of justice has publicly declared his insanity? Our author is perhaps right in answering this question in the negative; but he allows, as we have seen, that the public authorities ought to interfere. The practice on these points varies very much in different parts of France.

"In several departments it is only necessary to apply to the superintendants, in order to obtain admission for an insane person into the hospital, house, or asylum, especially set apart for these patients. In some places the authority of the mayor is necessary, because the establishment belongs to the corporation; in others the signature of the prefect is indispensable, because the establishment belongs to the department; whilst in a few departments the patient must be interdicted before his admission." (P. 84.)

M. Esquirol wishes the present system to be altered, but does not exactly state what he would recommend to be substituted. Not so the translator; he is for sweeping every one into the net: we shall catch the insane, and, as for the sane, let them get out again as well as they can. But he expresses himself upon this subject with such laudable frankness, that we must really disentangle his sentiments from the modest obscurity of a foot-note, and set them forth in the full glare of typographic emphasis.

"*It is very desirable that all obstacles to the admission of the insane into public and private asylums should be removed.* IT IS TRUE THIS CANNOT BE DONE WITHOUT RUNNING SOME RISK OF INCARCERATING PERSONS WHO ARE NOT FIT OBJECTS FOR CONFINEMENT; *but, when it is considered how many cases of suicide are continually coming before the public, which by confinement might have been prevented, and how many cases of insanity are rendered incurable by injudicious treatment at home, it can hardly be doubted but that great advantage would result from removing all unnecessary impediments to the confinement of the insane.*"

When you peruse this note, gentle reader, you will say "this was written by the keeper of a madhouse;" and so it was: you have guessed aright. But perhaps some of our youngest readers may inquire why the law has interposed what Mr. Liddell calls "numerous and vexatious obstacles to the admission of insane persons into houses licensed for their reception?" We will endeavour to answer this question. It sometimes happens that, in one family, a brother will run up enormous tavern-bills; in another, a retired merchant buys daubs at the price of Titians; in a third, a countess in her own right will drink spirits like a washerwoman; in a fourth, a young lady shows a decided partiality for the footman; and in a fifth, a maiden aunt, with 20,000*l.* in the three per cents. obstinately perseveres in living to an antediluvian age. In these, and a thousand similar instances, madness is the ever-ready cry of unscrupulous pride or avarice; and, unless the law looks with a stern jealousy on all accusations of insanity, we may see a man dragged to perpetual imprisonment whose sole crime consists in being rich, and wearing a straw hat in winter; or we might find the unworldly passions of the young and amorous characterized as insanity by their prudent parents, and the English madhouse playing the part of the continental convent. It is for these reasons that we think the present act defective, not because it interposes too many obstacles, but too few: its cobweb securities can be instantly annihilated by the conjoined signatures of two lads just fresh from their examination at the hall; and a man may find himself imprisoned by the fiat of practitioners to whose care no humane householder would commit a sick cat. The appointment of medical coroners, among other advantages, would bring with it that of having a competent authority to whose decision the public might appeal with confidence, in cases of doubtful insanity.

